# Unraveling Effects of miRNAs Associated with APR Leaf Rust Resistance Genes in Hybrid Forms of Common Wheat (*Triticum aestivum* L.)

**DOI:** 10.3390/ijms26020665

**Published:** 2025-01-14

**Authors:** Julia Spychała, Aleksandra Noweiska, Agnieszka Tomkowiak, Roksana Bobrowska, Katarzyna Szewczyk, Michał Tomasz Kwiatek

**Affiliations:** 1 Plant Breeding and Acclimatization Institute–National Research Institute in Radzików, 05-870 Błonie, Poland; m.kwiatek@ihar.edu.pl; 2 Department of Genetics and Plant Breeding, Poznań University of Life Sciences, 60-637 Poznań, Poland; agnieszka.tomkowiak@up.poznan.pl (A.T.); roksana.bobrowska@up.poznan.pl (R.B.); katarzyna.szewczyk1@up.poznan.pl (K.S.)

**Keywords:** adult plant resistance, leaf rust, *Lr46-Glu2*, target genes, small RNAs

## Abstract

The fungus *Puccinia triticina* Eriks (*Pt*) is the cause of leaf rust, one of the most damaging diseases, which significantly reduces common wheat yields. In *Pt*-resistant adult plants, an APR-type resistance is observed, which protects the plant against multiple pathogen races and is distinguished by its persistence under production conditions. With a more complete understanding of the molecular mechanisms underlying the function of APR genes, it will be possible to develop new strategies for resistance breeding in wheat. Currently, mainly APR genes, such as *Lr34*, *Lr46*, and *Lr67*, are principally involved in resistance breeding as they confer durable resistance to multiple fungal races occurring under different climatic and environmental conditions. However, the mechanisms underlying the defence against pathogens mediated by APR genes remain largely unknown. Our research aimed to shed light on the molecular mechanisms related to resistance genes and miRNAs expression, underlying APR resistance to leaf rust caused by *Pt*. Furthermore, the present study aimed to identify and functionally characterize the investigated miRNAs and their target genes in wheat in response to leaf rust inoculation. The plant material included hybrid forms of wheat from the F_2_ and BC_1_F_1_ generations, obtained by crossing the resistance cultivar Glenlea (CItr 17272) with agriculturally important Polish wheat cultivars. Biotic stress was induced in adult plants via inoculation with *Pt* fungal spores under controlled conditions. The RT-qPCR method was used to analyze the expression profiles of selected APR genes at five time points (0, 6, 12, 24, and 48 hpi). The results presented here demonstrate the differential expression of APR genes and miRNAs at stages of leaf rust development at selected timepoints after inoculation. We analyzed the expression of three leaf rust resistance genes, using different genetic backgrounds in F_2_ and BC_1_F_1_ segregation materials, in leaf tissues after *Pt* infection. Our goal was to investigate potential differences resulting from the genetic background found in different generations of hybrid forms of the same parental forms. Gene ontology analysis predicted 190 target genes for tae-miR5384-3p and 167 target genes for tae-miR9653b. Our findings revealed distinct expression profiles for genes, with the highest expression levels observed mainly at 6, 24, and 48 hpi. The candidate gene *Lr46-Glu2* displayed an upregulation, suggesting its potential involvement in the immune response against *Pt* infection.

## 1. Introduction

The global area under common wheat cultivation is more than 215 million hectares per year. Moreover, the scale of global wheat trade is larger than that of maize and rice combined [1]. Wheat grains are one of the most crucial sources of protein, providing approximately 20% of the calories consumed by people worldwide [2,3,4,5]. A disruption of these proportions could lead to a major crisis, i.e., insufficient food availability and economic changes resulting from the increased prices of wheat-containing products [6,7]. The growth of the world’s population is forcing suppliers to satisfy a growing demand for food. In order to meet these expectations, wheat production has to be increased by approximately 60% by 2050, which imposes a substantial challenge on breeders. Currently, the main directions of wheat breeding focus on high yields, high trade quality, and resistance to biotic and abiotic stresses [3,8,9]. To develop a sustainable and cost-effective strategy to control the spread of cereal diseases, it is therefore necessary to implement a multidisciplinary research approach involving breeders, farmers, geneticists, phytopathologists, and biotechnologists [10].

Worldwide wheat production is constantly exposed to diseases and pests that are evolving and spreading to new areas [11]. Climate change is favoring an increase in the incidence of diseases such as powdery mildew, stripe rust, leaf rust, and septoria tritici blotch, leading to an average of 20% damage to wheat yields each year [12]. Rust-causing fungi are biotrophic and are consequently entirely dependent on nutrient resources obtained from living host cells [13]. Rust fungi differ widely in the number of hosts they can infect; for example, wheat leaf rust (*Pt*) can only infect plants belonging to *Triticum* and *Aegilops* genera [14]. One of the current priorities in the industry is improved and climate-friendly agronomic practices, reducing the use of crop protection products [13]. Therefore, an understanding of the genetic variability of plant resistance, the evolutionary capacity of pathogens, and the choice of modern breeding methods are essential for successful resistance breeding. Common wheat (*Triticum aestivum* L.) is an allohexaploid species (2n = 6x = 42; AABBDD) derived from a dynamic domestication history [15]. The polyploidy of this species is the result of two rounds of hybridization: the first interspecific hybridization between the wheat-related *T. urartu* (2n = 2x = 14; AA) [16] and an undiscovered species related to *Aegilops speltoides* (genome BB), and the second between *T. turgidum* (2n = 4x = 28; AABB) and *A. tauschii* (2n = 2x = 14; DD) [17,18]. However, the origin of the B subgenome is still unclear. Currently, there is no diploid species whose chromosomes have high affinity for the B subgenome, and this suggests that the ancestral species is extinct or still unidentified [16]. Alternatively, there is a third possibility, which is the polyphyletic origin of subgenome B and its evolution at the polyploid level through multiple crosses with related species, such as *Ae. speltoides*, *Ae. bicornis*, *Ae. sharonensis*, *Ae. longissima*, and *Ae. searsii* [19,20]. In comparison to wheat’s wild relatives and its old cultivars, modern cultivars show increasingly lower genetic diversity, thereby also having a decreasing potential for disease-resistant genotypes. One of the strategies to overcome low genetic diversity in wheat is to exploit the gene pools present in native cultivars and wild ancestors of wheat [21,22]. Gene banks represent a huge potential that can be used in research and breeding applications to increase wheat resistance to biotic and abiotic stresses. Thus, by using closely related wild varieties to cross with elite crop cultivars, it is possible to introduce new genetic sources of desirable traits into breeding, especially genes that determine disease resistance. These genes can then be retained in subsequent hybrid generations through molecular-marker-assisted backcrossing [23]. Thereby, gene banks can be used to diversify resistance determinants, although the introgression of new alleles is a long and difficult process. Therefore, to accelerate the discovery, introgression, and pyramiding of resistance genes in resistance breeding, it is crucial to integrate as many tools as possible, most importantly advanced molecular biology techniques [24].

According to recent research, over 80 *Lr* genes and 14 additional leaf rust resistance genes have been identified in wheat. The above 14 genes were not assigned a new number in the *Lr* collection because they were not evaluated for allelism with known *Lr* genes to determine novelty [25]. There are two groups of resistance genes used in breeding wheat resistant to rust diseases. The first group, known as resistance genes (R major genes), confer specific resistance against one particular race of pathogen. Most of the identified *Lr* genes belong to this group [26]. In contrast, the second group, classified as APR resistance genes, is race-unspecific and often manifests itself as a “slow rusting” effect, which is associated with slower infection development, a longer latency period, and the formation of fewer uredinia. APR genes provide partial but durable resistance to pathogens [27]. The APR genes encode different proteins, such as ABC transporters, protein kinases, or hexose transporters, and these include *Lr34/Yr18/Pm38/Sr57*, *Lr46/Yr29/Pm39/Sr58*, and *Lr67/Yr46/Pm46/Sr55.*

The regulation of gene expression is enormously important, being essential for knowing and understanding the molecular mechanisms of resistance. In addition, researchers are increasingly turning their attention to the role of small non-coding RNAs (miRNAs, siRNAs), which play a significant role in the control of molecular processes. A more thorough understanding of the regulation and expression profile of genes related to RNA interference (RNAi) mechanisms and tolerance to abiotic or biotic stresses could lead to improved agronomic traits in many crops worldwide [28]. In plants, miRNA molecules are encoded by *MIR* genes, which are located in non-coding regions of the genome and are normally transcribed by RNA polymerase II [29]. miRNA are small, non-coding endogenous RNAs of 20-to-24 nucleotides (nt) that play a key role in regulating cellular processes. These small molecules can downregulate the protein levels of their target genes by repressing translation, cleaving the transcript, or inhibiting transcription [30,31]. The aim of the presented study was a multilevel analysis of the molecular mechanisms of resistance related to APR genes and miRNAs expression in response to *Pt* inoculation. In the experiment, we analyzed the expression of three leaf rust resistance genes, using different genetic backgrounds in F_2_ and BC_1_F_1_ segregation materials, in leaf tissues after *Pt* infection. Our goal was to investigate potential differences resulting from the genetic background found in different generations of hybrid forms of the same parental forms. Furthermore, the present study aimed to identify and functionally characterize the investigated miRNAs and their target genes in the common wheat genome using GO analysis.

## 2. Results

### 2.1. Identification of Molecular Markers Associated with APR Genes in Hybrid Forms

The use of molecular markers successfully identified selected *Lr* resistance genes in the wheat hybrid forms tested. The marker *csLV34*, anchored to the *Lr34* gene sequence, was used to verify the presence of the respective alleles (Table 1). According to the literature, the STS marker *csLV34* is highly reliable for the identification of *Lr34* [32]. If *Lr34* is present, a product of 150 bp will be obtained. The absence of the *Lr34* gene is indicated by the 229 bp product obtained. In a previous study, four hybrid forms were analyzed, a heterozygous system was obtained in the F_1_ generation [33], and only in Harenda × Glenlea form was a homozygote (+) obtained. In the absence of *Lr34*, a PCR product of 229 bp (−) was obtained. Further crosses resulted in hybrid forms of the F_2_ and BC_1_F_1_ generations, which were also subjected to selection using the above molecular markers in the presented work (Table 1). In these generations, the use of the *csLV34* marker allowed for the identification of 46% heterozygous (H) forms of the *Lr34* gene and 6% homozygous (+) forms (Table 1).

The *csLV46G22* marker linked to the *Lr46/Yr29* locus (E. Lagudah, unpublished data) was identified in all hybrid wheat forms of the obtained generations tested (Table 1). For *cfd23*, an amplification product of 211 bp was obtained, while for *cfd71*, a product of 214 bp was obtained. When the *cfd23* marker was used, the presence of the *Lr67* resistance determinant allele was confirmed in 55% of the hybrid forms of the F_2_ and BC_1_F_1_ (+) generations, while the *cfd71* marker allowed for identification of the gene in 62%. Amplicons of both flanking markers (*cfd23* and *cfd71*) [34] were identified in 35% of the hybrid forms of the F_2_ and BC_1_F_1_ (+) generations. Obtaining the above results allowed for a preliminary characterization of the genotypes and the selection of the obtained hybrid forms for further experiments, which included analysis of the expression of the aforementioned resistance genes and the candidate gene.

### 2.2. Observation and Evaluation of Infection Development by Pt

Observations of the plants after inoculation at the adult plant stage showed that the hybrids tested had low levels of infection by *Pt.* Based on the results of molecular marker identification, three plants were selected from each hybrid combination in which the presence of molecular markers was confirmed. The selected plants constituted biological replicates in the experiment. The evaluation of infection was performed at the adult plant stage (BBCH 39) using a rating scale according to COBORU (Research Centre For Cultivar Testing, Słupia Wielka, Poland (www.coboru.gov.pl/index_en; accessed on 9 September 2024)). The results of the infestation assessment are presented in Table 2.

### 2.3. Analysis of APR Gene Expression in BC_1_F_1_ and F_2_ Generations

**Figure 1:**
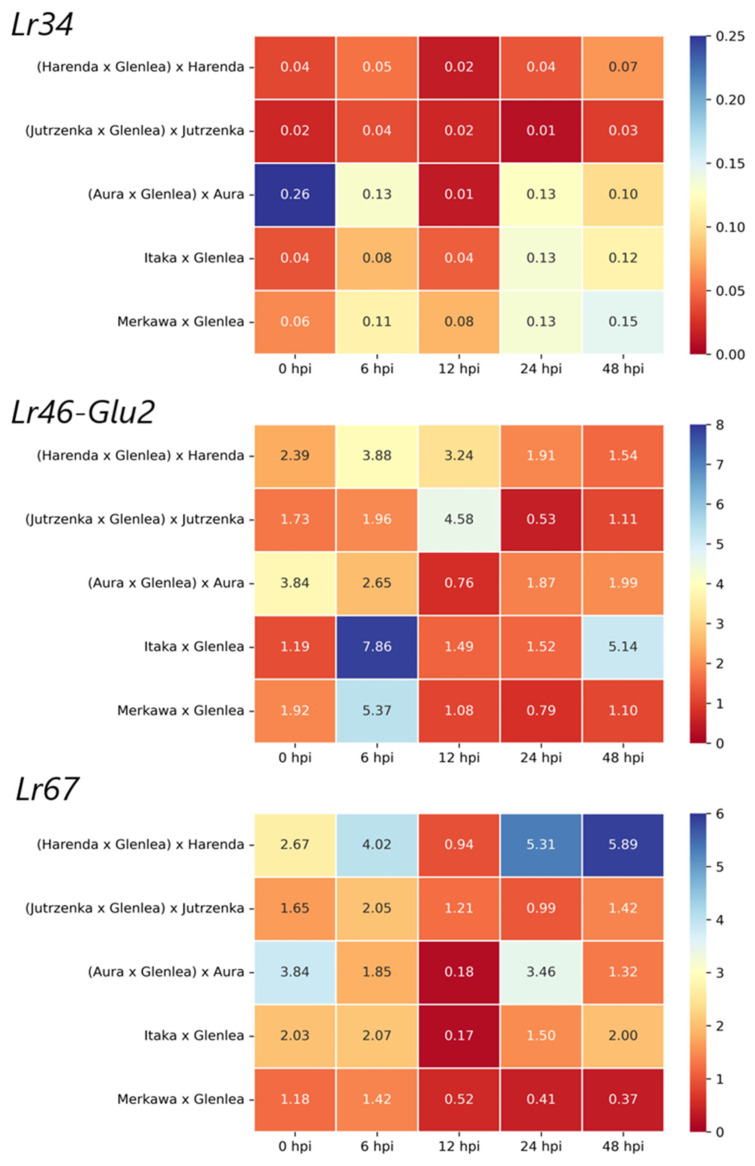
Heatmap plots showing the expression of *Lr34* and *Lr67* and the selected candidate gene *Lr46-Glu2* in leaf tissues after *Pt* infection. Expression analysis, using the RT-qPCR method, included wheat hybrid forms of BC_1_F_1_ and F_2_ generations at five time points (0, 6, 12, 24, and 48 hpi). Red indicates lower normalized expression, and blue indicates higher normalized expression.

The expression results of the four candidate genes for *Lr46*, presented in our previous studies [33], allowed us to select *Lr46-Glu2* as the gene with the highest expression after inoculation with *Pt*. Therefore, the gene expression analysis in hybrid wheat forms of generations BC_1_F_1_ and F_2_ included the three genes studied: *Lr34*, *Lr46-Glu2*, and *Lr67* (Figure 1). For the *Lr34* gene, as in previous years, it was characterized by low expression at all time points. However, an increase in expression could be observed in most of the hybrid forms tested, except (Harenda × Glenlea) × Harenda, (Jutrzenka × Glenlea) × Jutrzenka, which showed low expression at all time points. Interestingly, in (Aura × Glenlea) × Aura, an initial high expression level can be observed at 0 hpi. Thereafter, expression levels fluctuate but do not reach levels higher than before pathogen inoculation (Figure 1). Interestingly, the gene in both forms of the F_2_ generation, namely, Merkawa × Glenlea and Itaka × Glenlea, responded very similarly, reaching similar expression values (Figure 1).

The candidate gene *Lr46-Glu2* showed the highest expression in two forms of the F_2_ generation, namely, Merkawa × Glenlea and Itaka × Glenlea, reaching the highest values at 6 hpi (Figure 1). There was a decrease in expression at subsequent time points, but the Itaka × Glenlea hybrid form showed a renewed increase in *Lr46-Glu2* expression levels at 48 hpi (Figure 1). In contrast, the gene expression profile of the BC_1_F_1_ hybrid forms (Harenda × Glenlea) × Harenda and (Jutrzenka × Glenlea) × Jutrzenka was quite comparable, with the highest increase in *Lr46-Glu2* expression levels occurring at 12 hpi (Figure 1).

Analysis of *Lr67* expression showed that the response to inoculation varied widely among the hybrid forms tested (Figure 1). The most dynamic expression pattern was observed only in (Harenda × Glenlea) × Harenda, with a transient decrease in expression at 12 hpi. An interesting expression profile of this gene can also be observed in the second hybrid form from the BC_1_F_1_ generation, namely, (Aura × Glenlea) × Aura, where we observe a high level of expression even before inoculation (0 hpi). Then, a decrease in expression can be observed up to 12 h after inoculation and a dynamic increase in expression at 24 hpi (Figure 1). We also observed high basal expression of the F_1_ form of the Aura cultivar in our previous study [33], where high expression at 0 hpi was noted in all genes tested (*Lr34*, *Lr46-Glu2*, *Lr67*). Very similar expression patterns can be observed in F_1_ hybrid forms [33], with the exception of Jutrzenka × Glenlea.

Statistical analysis of the test genes, namely, *Lr34*, *Lr46-Glu2*, and *Lr67*, was also performed. The Kolmogorov–Smirnov test was used to test the null hypothesis that the set of normalized expression data for a particular gene and lineage comes from a normal distribution. This test was passed for all combinations of hybrid forms and genes except *Lr46-Glu2* in the Itaka × Glenlea hybrid form (*p*-value = 0.02397) (Supplementary Tables S1–S3). Levene’s Brown–Forsythe test was used to assess the equality of variance. The result of this test was positive for all tested combinations of hybrid forms and time points. To compare means, a two-sided Student’s *t*-test was performed (Supplementary Tables S1–S3). The stability of reference gene expression was statistically calculated, and cT values at each time point tested after inoculation were compared to cT values before pathogen inoculation (Supplementary Table S4).

### 2.4. Analysis of miRNA Expression of Wheat Hybrid Forms of BC_1_F_1_ and F_2_ Generations

**Figure 2:**
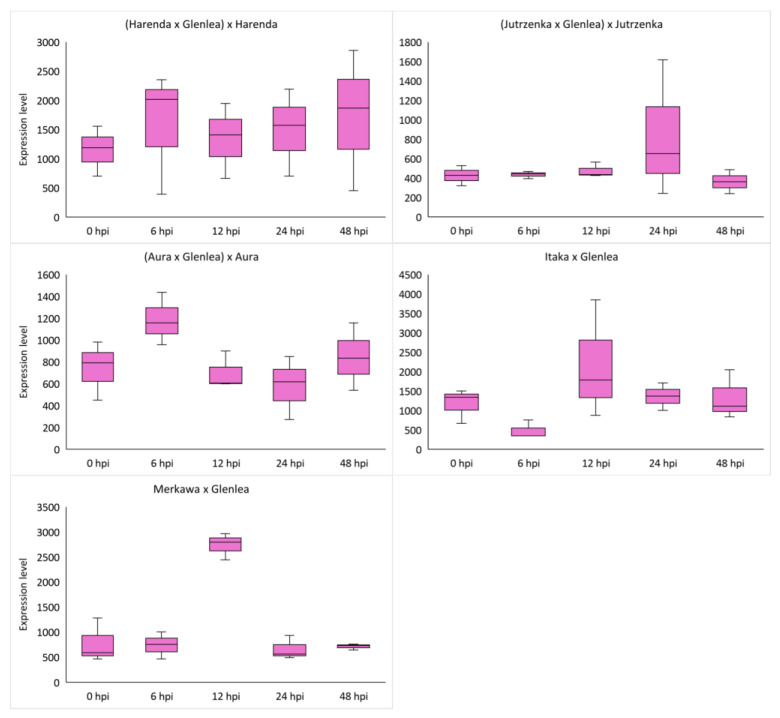
Box plots for tae-miR9653b expression at five timepoints after *Pt* inoculations. The ddPCR results show the numbers for the analyzed miRNAs during infection induced by *Pt*. Numbers 6, 12, 24, and 48 indicate hours after inoculation (hpi). The expression data shown were obtained from the analysis of three biological replicates, and each biological replicate had three technical replicates.

**Figure 3:**
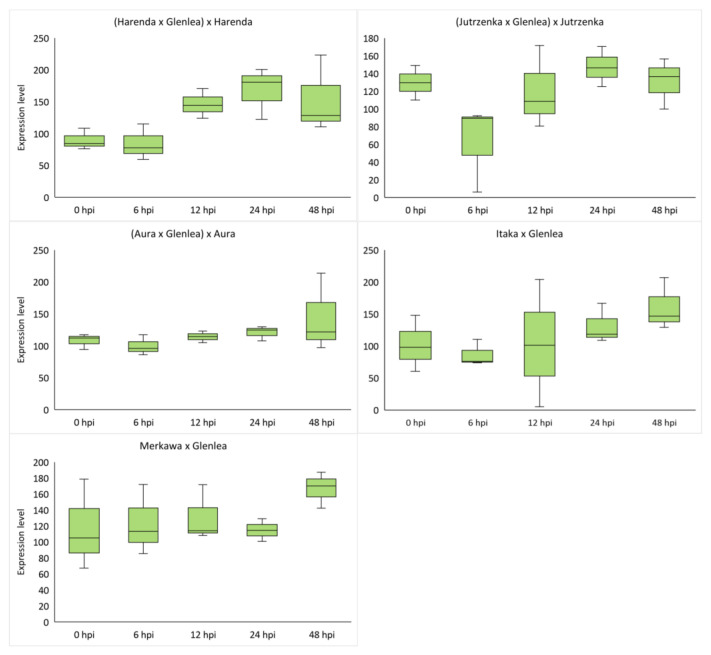
Box plots for tae-miR5384-3p expression at five timepoints after *Pt* inoculations. The ddPCR results show the droplet numbers for the analyzed miRNAs during infection induced by Pt. Numbers 6, 12, 24, and 48 indicate hours after inoculation (hpi). The expression data shown were obtained from the analysis of three biological replicates, and each biological replicate had three technical replicates.

The use of stem-loop RT-PCR and expression analysis via ddPCR was used to analyze the expression of miRNA molecules selected from databases as complementary to *Lr34* (tae-miR9653b; Table 3, Figure 2) and the candidate gene *Lr46-Glu2* (tae-miR5384-3p; Table 3, Figure 3). The obtained expression results of the selected genes were compared with the corresponding values for complementary miRNA molecules. Analysis of miRNA expression using ddPCR confirmed the potential role of tae-miR9653b in inhibiting the expression of the target gene—in this case, *Lr34*. Finally, in 24 and 48 hpi, when an increase in *Lr34* gene expression is observed, tae-miR9653b levels decrease. Such a tendency was observed in hybrid forms of the F_2_ generation: Itaka × Glenlea and Merkawa × Glenlea (Figure 2). Interestingly, the *Lr34* gene identified in Merkawa × Glenlea was present in the homozygous form (Table 1). The highest expression of tae-miR9653b molecules was observed in hybrid forms of the BC_1_F_1_ generation: (Aura × Glenlea) × Aura, Merkawa × Glenlea, and (Jutrzenka × Glenlea) × Jutrzenka at 6, 24, and 48 hpi, respectively (Figure 2). An analysis of the tae-miR5384-3p gene, which is complementary to this gene, found that its expression level fluctuated after inoculation in the four hybrid forms, eventually adopting higher values than the initial values before inoculation. Therefore, this may suggest that tae-miR5384-3p did not specifically repress the *Lr46-Glu2* gene since the expression of this gene increased after inoculation. The highest expression of tae-miR5384-3p molecules was also observed in hybrid forms of BC_1_F_1_ generation—(Harenda × Glenlea) × Harenda, (Jutrzenka × Glenlea) × Jutrzenka, and (Aura × Glenlea) × Aura—at 24 hpi and 48 hpi (Figure 3). Table 3 shows the averaged values of the obtained expression levels (copies/μL) of the analyzed miRNA molecules using the ddPCR method.

### 2.5. GO Results for Tested miRNAs

**Figure 4:**
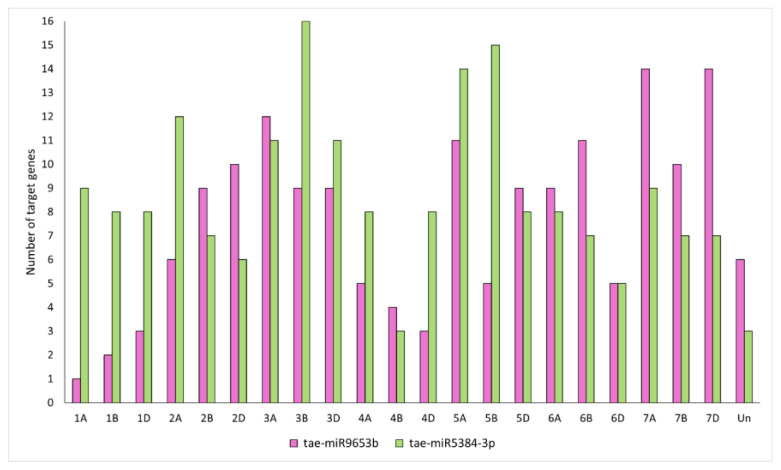
Number of predicted genes for specific wheat chromosomes for the miRNAs analyzed. “1A” and similar abbreviations indicate the name of the chromosomes of common wheat.

**Figure 5:**
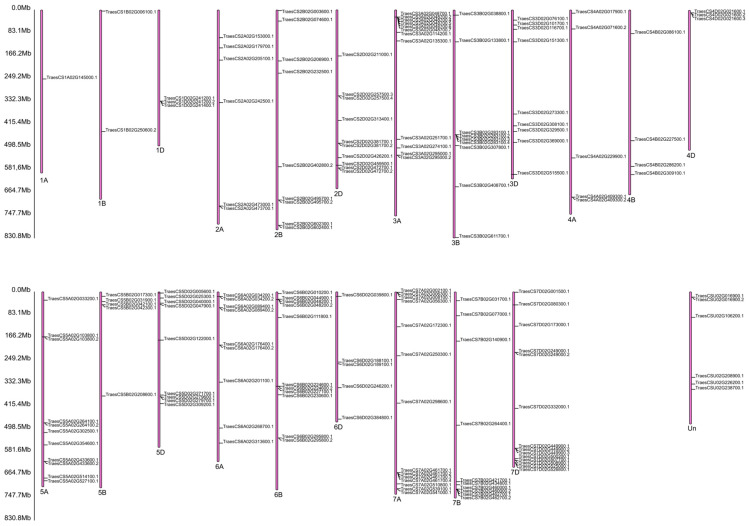
Localization of 167 target genes for tae-miR9653b on common wheat chromosomes. “1A” and similar indicate the name of the chromosome, and the abbreviation “Mb” next to the scale indicates the megabase unit referring to the physical length of the chromosomes.

**Figure 6:**
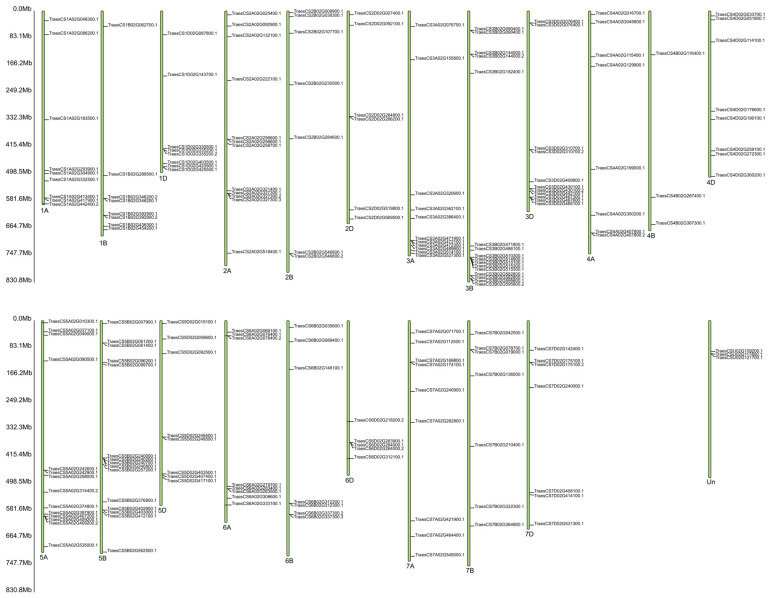
Localization of 190 target genes for tae-miR5384-3p on common wheat chromosomes. “1A” and similar indicate the name of the chromosome, and the abbreviation “Mb” next to the scale indicates the megabase unit referring to the physical length of the chromosomes.

**Figure 7:**
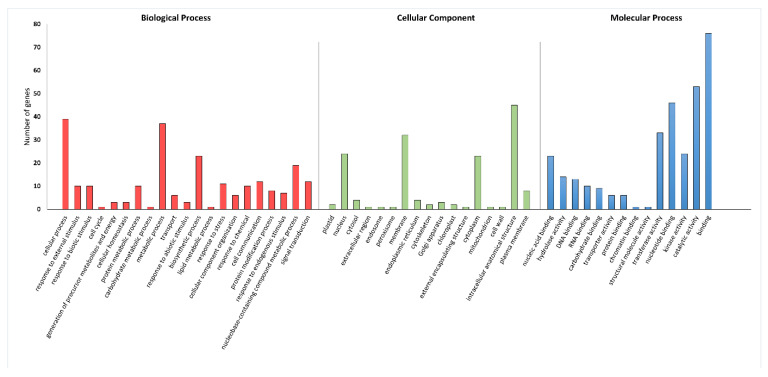
GO annotation of target genes for tae-miR9653b for three groups: biological process; cellular component; molecular function.

**Figure 8:**
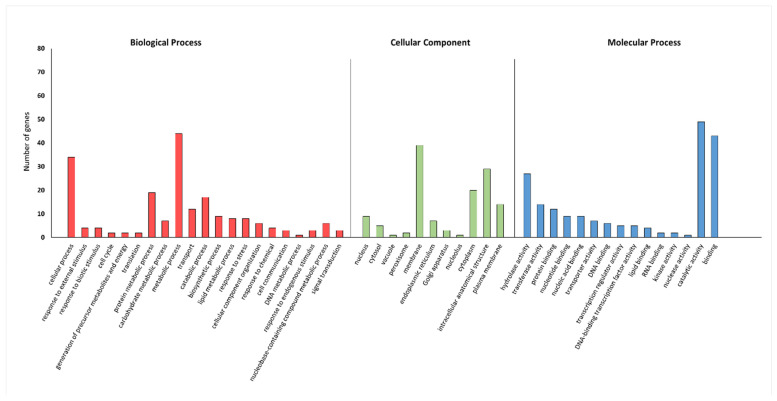
GO annotation of target genes for tae-miR5384-3p for three groups: biological process; cellular component; molecular function.

A gene ontology (GO)-based enrichment analysis was conducted to further investigate the potential role of the miRNAs studied in the wheat response to leaf rust infection (Figure 4). For miRNA tae-miR9653b, the most genes (14) were located on chromosomes 7A and 7D (Figure 4). On chromosome 7A, they were located in the distal part of the short and long arms; on chromosome 7D, they were mostly located in the subtelomeric region of the long arm (Figure 5). For the analyzed miRNA tae-miR5384-3p, 16 genes were identified on chromosome 3B (Figure 4), mainly located in the distal part of the long arm (Figure 6). The sequence of studied mature miRNAs was analyzed using psRNATarget software.(www.zhaolab.org/psRNATarget/; accessed on 9 September 2024). The 190 target genes for tae-miR5384-3p and 167 target genes for tae-miR9653b have been predicted in the outcomes (Supplementary Materials File S5, Figure 5 and Figure 6). GO analyses describe the functions, compartments, and biological processes in which genes are involved in a given organism, dividing them into three main domains: molecular function, biological process, and cellular component. The functional enrichment analysis of genes demonstrated the overall characteristics of candidate genes regarding GO functional entries (Figure 7 and Figure 8). Primary GO (level 2) analysis of the tae-miR9653b miRNA for biological processes revealed 21 functions, with the largest number of genes located within the cellular process (GO:0009987), metabolic process (GO:0008152), and biosynthetic process (GO:0009058) domains. For the cellular component domain, 17 functions were identified. In this domain, the greatest number of target genes were involved in the intracellular anatomical structure (GO:0005622), the membrane (GO:0016020), the nucleus (GO:0005634), and the cytoplasm (GO:0005737). For molecular process, 14 functions were observed, with most genes involved in binding (GO:0005488), catalytic activity (GO:0003824), nucleotide binding (GO:0000166), and transferase activity (GO:0016740) (Figure 7). In the GO analysis for tae-miR5384-3p in biological processes, the greatest number of genes were involved in the metabolic process (GO:0008152), the cellular process (GO:0009987), and the protein metabolic process (GO:0019538). For the cellular component aspect, the greatest number of genes were located in the membrane, the intracellular anatomical structure (GO:000562), the cytoplasm (GO:0005737), and the plasma membrane (GO:0005886). In biological processes, target genes mainly included catalytic activity (GO:0003824) and binding (GO:0005488) (Figure 8).

**Figure 9:**
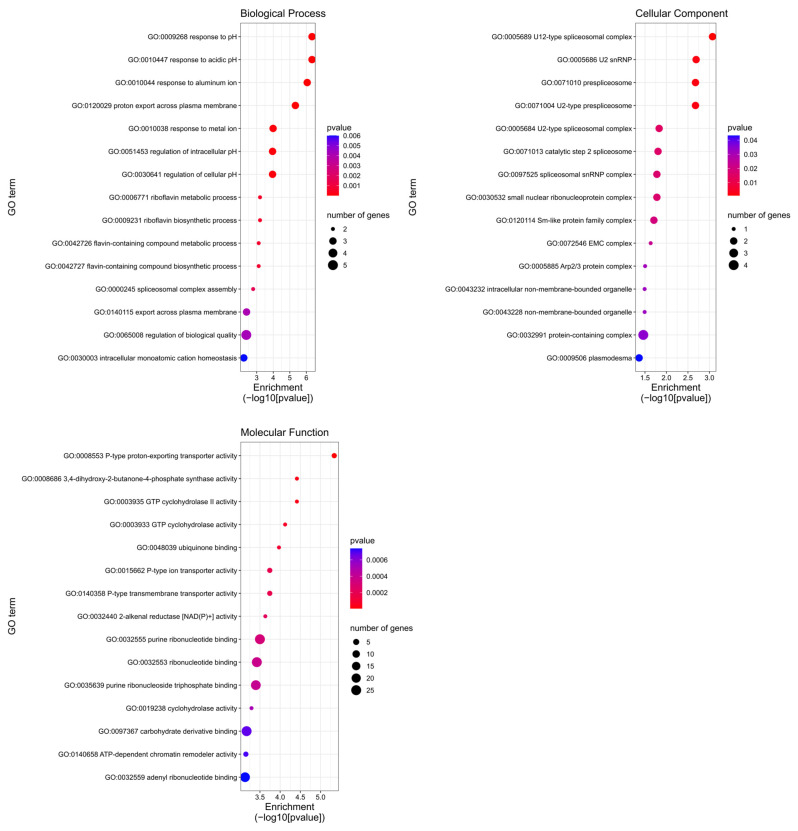
In–depth gene ontology (GO) pathway enrichment analysis for miRNA tae-miR9653b. The top 15 GO enrichment functions that were significantly enriched in biological processes, cellular components, and molecular functions were sorted by *p*-value.

**Figure 10:**
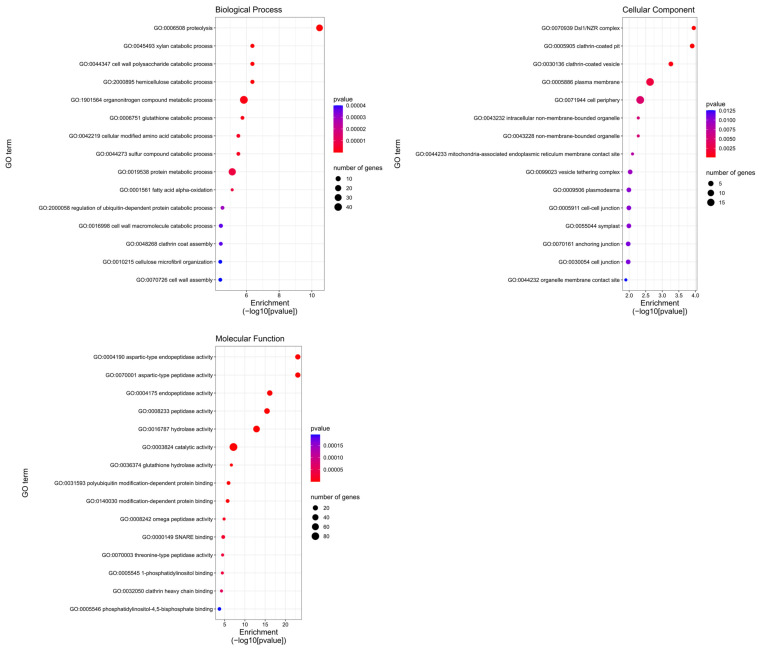
In–depth gene ontology (GO) pathway enrichment analysis for miRNA tae-miR5384-3p. The top 15 GO enrichment functions that were significantly enriched in biological processes, cellular components, and molecular functions were sorted by *p*-value.

More in-depth GO analysis of the predicted target genes was performed for GO enrichment. For this analysis, the *p*-value was used as a selection criteria (*p*-value < 0.05) (Supplementary Materials File S6). The *p*-value test permitted us to find for the tae-miR9653b molecule 56 GO terms related to biological processes, 19 GO terms related to the cellular component, and 59 Go terms related to the molecular function. Figure 9 displays the top 15 significantly enriched GO elements for the target genes of tae-miR9653b. The in-depth GO analysis, which was performed on a *p*-value basis, also showed functions related to the expression of the *Lr34* gene we studied, and included in the top 15 functions are purine ribonucleotide binding (GO:0032555), ribonucleotide binding (GO:0032553), adenyl ribonucleotide binding (GO:0032559), and carbohydrate derivative binding (GO:0097367). The GO analysis for the tae-miR5384-3p molecule uncovered 122 GO terms relating to biological processes, 20 relating to cellular components, and 78 relating to molecular functions (Supplementary Materials File S6). The top 15 significantly enriched GO terms for the tae-miR5384-3p target genes are shown in Figure 10. Furthermore, our in-depth analysis for the miRNA—*Lr46-Glu2* (TraesCS1B02G454200.1) in these GO terms includes the presented *Lr46-Glu2* candidate gene as a function of hydrolase activity (GO:0016787) and catalytic activity (GO:0003824) (Figure 10).

## 3. Discussion

In this paper, we present the results of *Lr34* and *Lr67* expression analyses in hybrid forms of wheat obtained by crossing a selected Glenlea variety with high-yielding Polish wheat cultivars. In addition, we analyzed the expression of miRNAs complementary to the studied APR genes and extended the bioinformatics analysis by performing gene ontology enrichment. The obtained hybrid forms included the F_1_ [33], F_2_, and BC_1_F_1_ generations. As in the previous study [33], in the F_1_ generation, the resistance genes *Lr34* and *Lr67* showed differential expression at selected time points. It should be noted that the *Lr34* gene is expressed at a relatively low level regardless of inoculation, while there is a rapid decrease in expression after inoculation (6 and 12 hpi). However, compared to the results described in our previous works, a similarity in the expression profile of *Lr34* in the donor form of Glenlea can be observed in the F_1_ hybrid forms tested, where the highest expression was observed at 24 and 48 hpi [33]. In the BC_1_F_1_ generation, an increase in *Lr34* gene expression can be observed in most of the hybrid forms tested, except (Harenda × Glenlea) × Harenda, (Jutrzenka × Glenlea) × Jutrzenka, which showed low expression at all time points. Notably, in the hybrid form (Aura × Glenlea) × Aura, we observed an initial high level of expression at 0 hpi. Thereafter, the expression level fluctuates but does not reach a level higher than before pathogen inoculation (Figure 1). Interestingly, the gene in both forms of the F_2_ generation, namely, Merkawa × Glenlea and Itaka × Glenlea, responded very similarly, reaching similar expression values (Figure 1). In contrast, *Lr67* expression showed that the response to inoculation was again very different between the F_2_ and BC_1_F_1_ hybrid forms tested (Figure 1). The most dynamic expression pattern was only observed in (Harenda × Glenlea) × Harenda, with a transient decrease in expression at 12 hpi. The interesting expression profile of this gene can also be observed in the second hybrid form of the BC_1_F_1_ generation, namely, (Aura × Glenlea) × Aura, where a high level of expression was observed even before inoculation (0 hpi), followed by a decrease in expression up to 12 hpi and a dynamic increase in expression at 24 hpi (Figure 1). Very similar expression patterns can be observed in F_1_ hybrid forms [33], with the exception of Jutrzenka × Glenlea.

According to the literature, the *Lr34* and *Lr67* genes present several similarities in that these genes determine resistance to multiple diseases in a non-race-specific manner and are responsible for encoding cellular transporters. The *Lr34* gene plays a key role in wheat breeding for disease resistance largely because it interacts with other resistance genes, resulting in effective and durable resistance [35]. Considering that the *Lr34* gene from hexaploid wheat functions as a transgene in other cereals, such as rice and barley, these diploid species with small genomes and fully annotated gene sets are highly suitable for an extensive study of *Lr34* function [24]. According to another recent study, the *Lr34* gene and *TaCOMT-3B* synergistically increase resistance to stripe rust [36]. Researchers in this study showed that wheat lines carrying the *Lr34res* allele presented thicker cell walls and increased resistance to fungal penetration compared to lines without *Lr34res*. The aim of the study presented by McCallum and Hiebert (2022) was to determine whether the *Lr67* gene also interacts with other resistance genes in a similar manner to *Lr34* [35]. To this end, six different doubled haploid lines were developed, which were characterized by the presence of the *Lr67* or *Lr34* gene together with a second resistance gene: *Lr13*, *Lr16*, or *Lr32*. The presence of the *Lr67* and *Lr34* genes significantly reduced the level of intensity of leaf rust growth, and the *Lr34* gene (unlike gene *Lr67*) showed a significant interaction with *Lr13*. Both genes interacted with *Lr16* and *Lr67* and had a significant interaction with the *Lr32* gene. This analysis shows a similar effect of the *Lr67* gene to that of *Lr34* in interacting with other genes to provide better resistance than single genes. Scientists suggest that though *Lr67* is not widely implemented in crops, it may play an important role in disease resistance [35]. Despite the introduction of the *Lr67* gene into many wheat varieties created by CIMMYT since the 1950s [37], it has not been found in Canadian wheat varieties to date. The researchers highlight that exploiting the potential of the *Lr67* gene in leaf rust countries could improve resistance in future wheat varieties [35].

On the basis of our previous results [33,38], we decided to select the candidate gene *Lr46-Glu2* for further studies involving the F_2_ and BC_1_F_1_ generations. The candidate gene *Lr46-Glu2* showed the highest expression in two forms of the F_2_ generation, namely, Merkawa × Glenlea and Itaka × Glenlea, reaching the highest values at 6 hpi (Figure 1). There was a decrease in expression at subsequent time points, but the Itaka × Glenlea hybrid form showed a renewed increase in *Lr46-Glu2* expression levels at 48 hpi (Figure 1). A similar expression pattern of this candidate gene was observed in previous experiments in the Glenlea cultivar [38] and in hybrid forms of the F_1_ generation [33]. In contrast, in the BC_1_F_1_ hybrid forms (Harenda × Glenlea) × Harenda and (Jutrzenka × Glenlea) × Jutrzenka, the gene expression profile was very similar, and the greatest increase in *Lr46-Glu2* expression levels occurred at 12 hpi (Figure 1).

The miRNAs regulate gene expression by binding to mRNA. The seed sequence is essential for miRNA binding to the mRNA transcript. The seed sequence or seed region is a conserved heptametric sequence that is mainly located at positions 2–7 from the 5′-end of the miRNA [39]. Hence, even if the base pairing of the miRNA and its target mRNA is not perfect, the “seed sequence” must be perfectly complementary. In summary, a miRNA can regulate gene transcription or translation in different ways through a seed sequence, a non-seed, or the entire sequence, thus providing the possibility of dual or multiple synergism in the regulation of one target gene by a single miRNA. As is known, endogenous miRNAs usually have a mild effect on their targets, but dual regulation of miRNAs can form complex networks and ultimately produce additive effects at the cellular and physiological levels. It is worth emphasizing that accurate miRNA modeling is essential, and the technologies for identifying miRNA targets have been greatly improved in recent years [39]. Currently, one of the goals of research in this field should be to determine the function of miRNA molecules in common wheat and its close relatives or ancestors, which may increase the chances of understanding species-specific miRNA molecules. Furthermore, the study of resistance mechanisms using molecular tools should find application in the breeding practice of key crop species [39].

In our series of studies of the tae-miR9653b molecule, we showed an expression profile suggesting its involvement in the *Pt*-induced biotic stress response [40,41]. In a study presented by Tomkowiak et al. (2023), the inoculation of wheat cultivars with *Pt* spores resulted in altered expression of tae-miR9653b in cultivars showing the presence of the resistance-determining *Lr34* allele [40]. The expression level of tae-miR9653b varied by cultivar and time point. At 6 hpi, there was a slight increase in the expression level of tae-miR9653b observed in all wheat reference cultivars, except for the control cultivar HN ROD. Most cultivars showed increased expression of this miRNA at 12, 24, and 48 h after inoculation. The fastest response of tae-miR9653 to inoculation was observed in the cultivar Myna‘S’ [40]. In the present study, our analysis of primary GO (level 2) for the tae-miR9653b for biological processes revealed 21 functions, with the largest number of genes located within the cellular process (GO:0009987), metabolic process (GO:0008152), and biosynthetic process (GO:0009058) domains. For the cellular component domain, 17 functions were identified. The in-depth GO analysis, which was performed on a *p*-value basis, also showed that the functions related to the expression of the *Lr34* gene we studied included in the top 15 functions purine ribonucleotide binding (GO:0032555), ribonucleotide binding (GO:0032553), adenyl ribonucleotide binding (GO:0032559), and carbohydrate derivative binding (GO:0097367).

Previous studies have shown that tae-miR9653b and tae-miR5384-3p may be involved in immune responses to both biotic and abiotic stresses. A study presented by Li et al. (2017) examined the expression of a number of different miRNAs, including tae-miR9653b, after wheat exposure to phenanthrene [42], and it was shown that the expression of the tae-miR9653b studied was decreased after the treatment of plants with phenanthrene [42]. In contrast, in a study on drought stress in common wheat [43], the authors considered the role of miRNA molecules in drought response. One of the three miRNAs repressed by drought in the drought-tolerant variety was tae-miR9653 (including miR9653a and miR9653b) [43]. In contrast, in the susceptible genotype, tae-miR9653b expression was increased, while tae-miR9653a expression levels remained unchanged. Unfortunately, knowledge of the role of tae-miR9653b in plant responses to biotic stresses is still limited, although tae-miR9653b has been shown to respond positively to *Fusarium graminearum* infection in common wheat lines carrying the FHB resistance locus in chromosome 2DL [44].

The tae-miR5384-3p molecule, the expression of which was studied in this paper, was found to fluctuate in its expression level after inoculation in the four hybrid forms, eventually adopting values lower or equal to the initial values before inoculation. Therefore, as gene expression increased after inoculation, this may suggest that tae-miR5384-3p did not specifically repress the *Lr46-Glu2* gene at the time points tested. Analyzing the tae-miR5384-3p molecule complementary to this gene, its expression level was found to increase after inoculation in four hybrid forms of the BC_1_F_1_ generation—(Harenda × Glenlea) × Harenda, (Jutrzenka × Glenlea) × Jutrzenka, and (Aura × Glenlea) × Aura—and an increase in tae-miR5338-3p was observed at 24 and 48 hpi. The presented GO analysis for the tae-miR5384-3p molecule uncovered 122 GO terms relating to biological processes, 20 relating to cellular components, and 78 relating to molecular functions. Furthermore, our in-depth analysis for the miRNA—*Lr46-Glu2* (TraesCS1B02G454200.1) in these GO terms includes the presented *Lr46-Glu2* candidate gene as a function of hydrolase activity (GO:0016787) and catalytic activity (GO:0003824). Literature reports have determined that the tae-miR5384-3p molecule can inhibit a number of genes in the *TaFAB2* subfamily, which are associated with the formation of unsaturated fatty acids that play a role in plant development and in response to biotic and abiotic stresses [45]. In another study, the tae-miR5384-3p molecule showed a very high increase in expression after treating wheat seedlings with a chitosan suspension [42]. Such a phenomenon may relate to changing defense mechanisms in the early and late stages of plant responses to stresses. On the other hand, Gidhi’s team (2022) conducted sequence analysis, revealing the involvement of tae-miR5384-3p (as well as tae-miR164 and tae-miR9679-5p) in targeting the *TaAFB6* gene (TraesCS5A02G281100), which encodes the *AUXIN SIGNALING F-BOX* (*AFB*) protein [46]. This suggested the possibility of post-transcriptional regulation of *TaAFB*-type gene expression. The researchers analyzed the expression of selected *AFB* genes in response to *Pt* spore inoculation by taking wheat leaf sections at six time points (0, 12, 48, 120, and 168 hpi). Some of the *AFB* genes analyzed showed highest expression at 12 and 48 hpi in both susceptible and resistant wheat lines. All known *TaAFB*-type genes are involved in the auxin-activated signaling pathway that leads to the ubiquitination process [46].

It is worth highlighting that the expression analyses presented here addressed gene expression patterns in various wheat hybrid forms obtained in F_2_ and BC_1_F_1_ generations, while their genetic background and specific resistance mechanisms are not precisely known. Investigating the evolutionary history of these varieties could provide a context for interpreting the observed expression patterns of the resistance and candidate genes studied. Future research should address the functional validation of the candidate genes mentioned, including, but not limited to, gene knockout and overexpression analysis. In addition, the integration of transcriptomic, proteomic, and metabolomic data can provide a more comprehensive understanding of the molecular mechanisms underlying *Pt* resistance. This multi-omics approach enables the identification of key pathways and regulatory networks involved in plant immune responses [47].

## 4. Materials and Methods

### 4.1. Crossbreeding Between Wheat Cultivars and Conducting Experiments in a Controlled Environment

**Figure 11:**
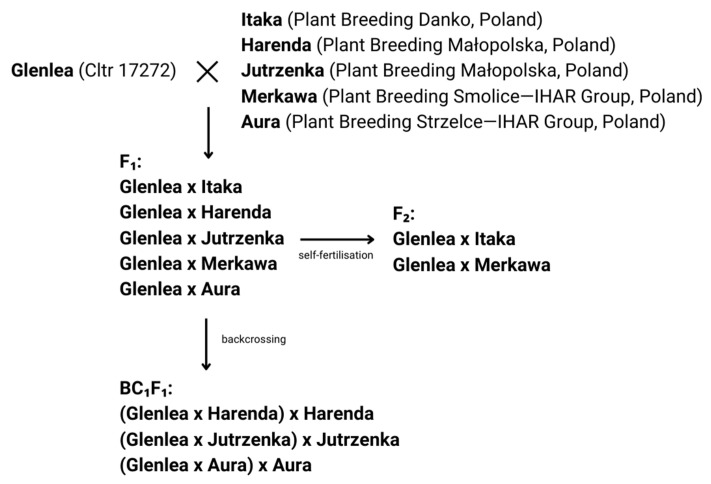
Diagram of crossbreeding conducted and the obtained hybrid forms of F_1_, F_2_, and BC_1_F_1_ generations.

The plant material consisted of five hybrid combinations from the F_2_ and BC_1_F_1_ generations obtained by crossing a Glenlea cultivar, obtained from the National Small Grains Collection of the United States Department of Agriculture—Agricultural Research Service (USDA-ARS) (Aberdeen, WA, USA), with economically important Polish spring wheat cultivars from plant breeding companies (Figure 11). The experiment was set up in a phytotron chamber with a system for maintaining and controlling constant temperature and humidity at the Wielkopolskie Centrum Zaanwansowanych Technologii (WCZT), 10 University of Poznań Street, 61-614 Poznań, Poland. Seedlings of hybrid forms were grown in the phytotron chamber at 22 °C and 17 °C (day and night, respectively), with a relative humidity of 60–70%, a 16 h photoperiod, and an illumination intensity of 250–300 μmol m^−2^ s^−1^.

### 4.2. Isolation of gDNA from Wheat Leaf Tissue

To confirm the presence of resistance-conditioning alleles in the hybrid forms tested, genomic DNA was extracted, and a PCR reaction was performed. DNA was isolated from leaves of 10-day-old seedlings (BBCH 13) using the GeneMATRIX Plant and Fungi DNA Purification Kit (EURx Ltd., Gdańsk, Poland) according to the procedure provided by the manufacturer. DNA concentration and quality were determined using a NanoDrop spectrophotometer at 260 nm. Samples were diluted with Elution buffer (EURx Ltd., Gdańsk, Poland) to a uniform concentration of 50 ng/µL.

### 4.3. Identification of Molecular Markers Associated with APR Genes

Polymerase chain reaction (PCR) was used to confirm the presence of alleles associated with the *Lr34*, *Lr46*, and *Lr67* genes. The marker *csLV34*, anchored to the sequence of the *Lr34* gene, was used to verify the presence of the respective alleles [48]. The marker *csLV46G22* linked to the *Lr46/Yr29* locus (E. Lagudah, unpublished data) was used to identify *Lr46*. Molecular flanking SSR markers, *cfd23* and *cfd71* [34], were used to confirm the presence of the *Lr67* gene. The PCR reaction was performed in a volume of 20 µL of reaction mixture, consisting of 1 µL of two primers (10 µM) (Merck-Sigma Aldrich, Burlington, MA, USA) and 12.5 µL of FastGene Optima HotStart ReadyMix (NIPPON Genetics Europe GmbH, Düren, Germany), which contained FastGene Optima DNA polymerase mix (0.2 U per 1 µL reaction), FastGene Optima buffer, dNTPs (0.4 mM), MgCl_2_ (4 mM), and stabilizers. The temperature profile of the reaction was as follows: initial denaturation at 94 °C for 5 min, followed by 35 cycles (denaturation at 94 °C for 45 s; primer attachment at 60 °C for 30 s; strand extension at 72 °C for 1 min), followed by the final strand extension for 7 min at 72 °C and storage at 4 °C. Amplification products using the *csLV46G22* marker were digested with the restriction enzyme *BspEI* (Thermo Fisher Scientific, Waltham, MA, USA) at 37 °C for 1 h (Lagudah, private correspondence, 2020). A Labcycler thermocycler (SensoQuest GmbH, Göttingen, Germany) was used to perform the digestion reaction. The resulting PCR products were analyzed using a 2% agarose gel (Bioshop, Canada Inc., Burlington, ON, Canada) in 1x TBE buffer (Bioshop, Canada Inc., Burlington, ON, Canada) with the addition of 7 µL Midori Green Advanced DNA Stain (Nippon Genetics Europe, Düren, Germany). The results of the electrophoresis were photographed under UV light using a GelDoc XR UV Molecular Imager system with Image Lab software v5.1 (Bio-rad Laboratories, Inc., Hercules, CA, USA).

### 4.4. Inoculation of Adult Plants with Pt Spores

After reaching the adult plant stage (flag leaf stage, BBCH 39), biotic stress in hybrid forms was induced via inoculation with spores of the fungus *P. triticina*. The inoculation material was a mixture of *Pt* isolates collected from infected field experiments owned by Polish breeding companies. Before inoculation, the viability of *Pt* spores was assessed under a microscope. Hybrid wheat plants were inoculated by spraying whole plants with urediniospores at a concentration of approximately 5 × 10^5^ spores/mL, suspended in sterile, nuclease-free water with Tween 20.

### 4.5. Observation and Evaluation of Pt Infection

The evaluation of infection included only selected plants in which the presence of molecular markers was confirmed. The selected plants constituted biological repeats in the experiment. Infection progression was assessed 10 days after *Pt* inoculation. To assess the stage of infection of the plants, a scale according to COBORU (Research Centre For Cultivar Testing, Słupia Wielka, Poland; www.coboru.gov.pl/index_en; accessed on 9 September 2024) was used: 1—complete infection; 9—no infection.

### 4.6. Total RNA Isolation and Reverse Transcription Reaction (cDNA Synthesis)

Isolation of total RNA from different leaf tissue samples collected at three biological replicates and time points (0, 6, 12, 24, and 48 hpi) was performed using the Maxwell RSC Plant RNA Isolation Kit (Promega, Madison, WI, USA) harnessing automated station Maxwell® RSC 48 Instrument (Promega). During RNA isolation, samples were treated with DNAase according to the manufacturer’s recommendations. The concentration and purity of isolated total RNA was measured using a NanoDrop spectrophotometer in the A260/A280 absorbance range. Synthesis of cDNA was performed with 1 µg RNA using the iScript Reverse Transcription Supermix for RT-qPCR kit (Bio-Rad, Hercules, CA, USA) according to the manufacturer’s protocol. The temperature profile of the cDNA synthesis reaction was as follows: pre-incubation for 5 min at 25 °C, reverse transcription for 60 min at 46 °C, inactivation of reverse transcriptase for 1 min at 95 °C, cooling to 4 °C, and storage at −20 °C.

### 4.7. Quantitative Gene Expression Analysis Using RT-qPCR

A two-step PCR protocol was exploited using iTaq Universal SYBR Green Supermix (Bio-Rad, Hercules, CA, USA) and the CFX Connect Real-Time PCR Detection System (Bio-Rad, Hercules, CA, USA). Each RT-qPCR experiment performed consisted of three biological repeats and three technical repeats, the results of which were averaged. In addition, a no template control (NTC), i.e., a negative control without a cDNA template, was also performed for each 96-well plate tested. The composition of the RT-qPCR reaction mixture was as follows: iTaq supermix—5 μL; forward and reverse primers (10 μM)—0.5 μL, each 3 μL of nuclease-free water; and cDNA template—1 μL. Following MIQE recommendations for the validity of RT-qPCR analyses, three biological repeats were used for each gene type at each time point for a given wheat cultivar or hybrid form, and three technical repeats were prepared for each [49]. Standard curves and reaction efficiency (%E) for tested and referenced genes were included according to earlier protocols [38,41]. Calculations of ∆∆Cq included both reference genes using CFX Maestro (Bio-Rad, Hercules, CA, USA).

### 4.8. miRNA Isolation and Reverse Transcription Reaction (Stem Loop RT-PCR)

In the present study, we analyzed the expression of miRNAs associated with the *Lr34* gene and the candidate gene for the *Lr46* locus. For the *Lr34* gene, primers for tae-miR9653b were used. For the *Lr46-Glu2* candidate gene, the expression of tae-miR5384-3p was analyzed. Available databases for common wheat and literature data did not show miRNA molecules complementary to the *Lr67* gene. The coding sequences of the miRNA target genes studied were downloaded from the Ensembl Plants database and analyzed in the psRNATarget online database. The sequences of the miRNA molecules analyzed were found in the miRBase database and downloaded in FASTA format. Primers for miRNA reverse transcription and ddPCR reactions were designed using the OligoAnalyzer™ Tool from the Integrated DNA Technologies website, following early protocols [38,41].

Leaf tissue for miRNA isolation was collected analogously to the RNA isolation protocol. The mirVana miRNA Isolation Kit from ThermoFisher Scientific and a phenol–chloroform mixture were used for miRNA isolation. The miRNA isolation was carried out according to the protocol provided by the manufacturer. The obtained samples were stored in a cryofreezer (−80 °C) to avoid possible degradation. The miRNA fraction was then subjected to reverse transcription reaction using SuperScript IV Reverse Transcriptase (Thermo Fisher Scientific, Waltham, MA, USA), employing specific designed primers according to the protocol developed by Varkonyu-Gasic et al. (2007) [50]: incubation at 16 °C for 30 min; 60 cycles of 30 °C for 30 s, 42 °C for 30 s, and 50 °C for 1 s; incubation at 85 °C for 5 min.

### 4.9. ddPCR Expression Analysis

To quantify the number of miRNA molecules in plant samples, a ddPCR mixture, consisting of 10 µL ddPCR SuperMix Eva Green, primers (200 nM), template (reverse-transcribed elongated miRNA), and RNase-free water, was used. A 20 µL reaction mixture was used to generate reaction droplets in an 8-well cassette using a QX100 droplet generator (Bio-Rad Laboratories, Inc., Hercules, CA, USA). The droplets were carefully transferred to a 96-well ddPCR plate and heat-sealed using plastic heat-film coverslips (Bio-Rad Laboratories, Inc., Hercules, CA, USA). Amplification was then performed in a T100 PCR thermocycler (Bio-Rad Laboratories, Inc., Hercules, CA, USA) according to the previous protocols [38,41]. The resulting PCR products were quantitatively analyzed in a QX100 droplet reader (Bio-Rad Laboratories, Inc., Hercules, CA, USA). Data analysis was performed using QuantaSoft software version 1.7 (Bio-Rad Laboratories, Inc., Hercules, CA, USA). Positive droplets containing amplification products were distinguished from negative droplets by setting the fluorescence amplitude threshold to the lowest value of the positive droplet cluster.

### 4.10. Statistical Analysis

The Kolmogorov–Smirnov test was used to test the null hypothesis that a set of normalized expression values for a particular gene and cultivar indicates a distribution close to normal. This test was performed for all gene × genotype combinations. For verification with respect to homogeneity of variance, the Levene’s variant Brown–Forsythe test was performed. For comparison of means, the Student’s *t*-test (two-sided) was performed. For each gene tested, expression values at each time point tested were compared to expression before inoculation with the spore mixture. Normalized gene expression was presented using heat maps. To test the stability of the expression of the reference genes, comparisons were made between the cT values at each tested time point after inoculation relative to the cT values before pathogen inoculation. Statistical analyses were performed using Excel 2021 and Python 3.11.7.

### 4.11. Target Gene Prediction for Selected miRNAs

To analyze the miRNA target genes, an online tool psRNATarget (www.zhaolab.org/psRNATarget/; accessed on 9 September 2024) was used [51]. The software applies a predefined score scheme based on the “seed” region, examining complementary pairing and evaluating miRNA target accessibility based on the energy required to open the secondary structures. Target prediction was carried out by querying mature wheat miRNA sequences towards “*Triticum aestivum* (bread wheat), cDNA, EnsemblPlant, release 43” under default-parameters Schema V2 (release 2017). Gene ontology (GO) analyses of the predicted target genes were conducted using GO (www.geneontology.org (accessed on 9 September 2024)). An analytical tool from the PANTHER Classification System (https://pantherdb.org/ (accessed on 9 September 2024)) was used, which is continuously updated with GO annotations. GO enrichment plots and gene expression heat maps were created using Phyton 3.11.7. and TBtools v2.121 [52]. An in-depth enrichment analysis bubble chart was constructed for visualization by SRPLOT website (www.bioinformatics.com.cn/srplot; accessed on 9 September 2024).

## 5. Conclusions

Our findings revealed distinct expression profiles for different genes, with the highest expression levels observed at 6, 24, and 48 hpi. Candidate gene *Lr46-Glu2* displayed an upregulation, suggesting its potential involvement in the immune response against *Pt* infection. As a result of the analyses of 10 candidate genes for *Lr46* locus obtained in previous generations, we decided to select *Lr46-Glu2* for the present study. In the two BC_1_F_1_ forms (Harenda and Jutrzenka), the gene expression profiles were very similar, with the increase in *Lr46-Glu2* expression levels at 12 hpi. The *Lr46-Glu2* gene showed the highest expression (6 hpi) in two F_2_ forms (Merkawa and Itaka). Analysis of *Lr67* expression showed that the response to inoculation varied widely. The *Lr34* gene was characterized by low expression at all time points. However, an increase in expression was observed in most of the hybrid forms tested, except Harenda and Jutrzenka. Furthermore, we observed potential intriguing interactions between APR genes expression and the associated miRNAs, which highlight the complex regulatory networks underlying *Pt* resistance. This study confirmed the potential role of tae-miR9653b in inhibiting *Lr34* target gene expression. Gene ontology analysis predicted 190 target genes for tae-miR5384-3p and 167 target genes for tae-miR9653b. The in-depth GO analysis, which was performed on a *p*-value basis, also showed functions related to the expression of the *Lr34* and *Lr46-Glu2* genes we studied. For tae-miR9653b, the most dynamic expression pattern was observed in Harenda hybrid form with the highest expression at 48 hpi and a temporary decrease at 12 hpi. As a result of the analysis of tae-miR5384-3p, it was found that its expression level fluctuated in the four hybrid forms, eventually adopting values lower than or equal to the initial values before inoculation. In contrast, tae-miR9653b levels decrease at 24 hpi to 48 hpi, when an increase in *Lr34* gene expression is observed. However, the results may suggest that tae-miR5384-3p did not specifically repress the *Lr46-Glu2* gene. Our presented study underscores the importance of further investigations to unravel the intricacies of wheat defense mechanisms against *Pt* and to facilitate the development of novel strategies for *Pt* resistance breeding.

## Figures and Tables

**Table 1:** Identification of molecular markers associated with the resistance genes studied in wheat hybrid forms of wheat from the F_2_ and BC_1_F_1_ generations. The grey color indicates the hybrid forms of wheat that have been selected and included in further molecular analyses.

No.	Plant Name	Breeding Company	Hybrid form BC_1_F_1_/F_2_	*Lr34* (*csLV34*)	*Lr46* (*csLV46G22*)	*Lr67* (*cfd23*)	*Lr67* (*cfd71*)
1.	H × G 1 I	Małopolska HR	(Harenda × Glenlea) × Harenda	H	+	+	-
2.	H × G 1 II	Małopolska HR	(Harenda × Glenlea) × Harenda	-	+	-	-
3.	H × G 1 III	Małopolska HR	(Harenda × Glenlea) × Harenda	H	+	-	+
4.	H × G 1 IV	Małopolska HR	(Harenda × Glenlea) × Harenda	H	+	-	-
5.	H × G 2 I	Małopolska HR	(Harenda × Glenlea) × Harenda	-	+	-	-
6.	H × G 2 II	Małopolska HR	(Harenda × Glenlea) × Harenda	-	+	-	+
7.	H × G 2 III	Małopolska HR	(Harenda × Glenlea) × Harenda	H	+	-	+
8.	H × G 2 IV	Małopolska HR	(Harenda × Glenlea) × Harenda	-	+	-	-
9.	H × G 3 I	Małopolska HR	(Harenda × Glenlea) × Harenda	-	+	-	-
10.	H × G 3 II	Małopolska HR	(Harenda × Glenlea) × Harenda	H	+	-	-
11.	H × G 3 III	Małopolska HR	(Harenda × Glenlea) × Harenda	-	+	+	-
12.	H × G 3 IV	Małopolska HR	(Harenda × Glenlea) × Harenda	H	+	-	+
13.	J × G 1 I	Małopolska HR	(Jutrzenka × Glenlea) × Jutrzenka	H	+	+	-
14.	J × G 1 II	Małopolska HR	(Jutrzenka × Glenlea) × Jutrzenka	H	+	+	-
15.	J × G 1 III	Małopolska HR	(Jutrzenka × Glenlea) × Jutrzenka	H	+	+	-
16.	J × G 2 I	Małopolska HR	(Jutrzenka × Glenlea) × Jutrzenka	-	+	-	-
17.	J × G 2 II	Małopolska HR	(Jutrzenka × Glenlea) × Jutrzenka	-	+	-	-
18.	J × G 2 III	Małopolska HR	(Jutrzenka × Glenlea) × Jutrzenka	-	+	-	-
19.	J × G 3 I	Małopolska HR	(Jutrzenka × Glenlea) × Jutrzenka	-	+	-	-
20.	J × G 3 II	Małopolska HR	(Jutrzenka × Glenlea) × Jutrzenka	H	+	+	+
21.	J × G 3 III	Małopolska HR	(Jutrzenka × Glenlea) × Jutrzenka	-	+	-	-
22.	J × G 3 IV	Małopolska HR	(Jutrzenka × Glenlea) × Jutrzenka	H	+	+	-
23.	17 I	Strzelce HR	(Aura × Glenlea) × Aura	-	+	+	+
24.	17 II	Strzelce HR	(Aura × Glenlea) × Aura	H	+	+	+
25.	17 III	Strzelce HR	(Aura × Glenlea) × Aura	H	+	+	+
26.	17 IV	Strzelce HR	(Aura × Glenlea) × Aura	-	+	+	+
27.	17 V	Strzelce HR	(Aura × Glenlea) × Aura	H	+	+	+
28.	18 I	Strzelce HR	(Aura × Glenlea) × Aura	-	+	+	+
29.	18 II	Strzelce HR	(Aura × Glenlea) × Aura	H	+	+	+
30.	18 III	Strzelce HR	(Aura × Glenlea) × Aura	H	+	+	+
31.	18 IV	Strzelce HR	(Aura × Glenlea) × Aura	H	+	+	+
32.	21 I	Strzelce HR	(Aura × Glenlea) × Aura	H	+	+	+
33.	21 II	Strzelce HR	(Aura × Glenlea) × Aura	H	+	+	+
34.	21 III	Strzelce HR	(Aura × Glenlea) × Aura	H	+	+	+
35.	21 IV	Strzelce HR	(Aura × Glenlea) × Aura	-	+	+	+
36.	21 V	Strzelce HR	(Aura × Glenlea) × Aura	-	+	-	+
37.	22 I	Strzelce HR	(Aura × Glenlea) × Aura	-	+	+	+
38.	22 II	Strzelce HR	(Aura × Glenlea) × Aura	-	+	+	+
39.	22 III	Strzelce HR	(Aura × Glenlea) × Aura	H	+	-	+
40.	22 IV	Strzelce HR	(Aura × Glenlea) × Aura	-	+	-	+
41.	22 V	Strzelce HR	(Aura × Glenlea) × Aura	H	+	-	+
42.	24 I	Strzelce HR	(Aura × Glenlea) × Aura	-	+	-	+
43.	24 II	Strzelce HR	(Aura × Glenlea) × Aura	-	+	-	+
44.	24 III	Strzelce HR	(Aura × Glenlea) × Aura	-	+	-	+
45.	24 IV	Strzelce HR	(Aura × Glenlea) × Aura	H	+	-	+
46.	24 V	Strzelce HR	(Aura × Glenlea) × Aura	-	+	-	+
47.	25 I	Strzelce HR	(Aura × Glenlea) × Aura	-	+	-	+
48.	25 II	Strzelce HR	(Aura × Glenlea) × Aura	-	+	-	+
49.	25 III	Strzelce HR	(Aura × Glenlea) × Aura	H	+	-	+
50.	25 IV	Strzelce HR	(Aura × Glenlea) × Aura	-	+	-	+
51.	25 V	Strzelce HR	(Aura × Glenlea) × Aura	H	+	+	+
52.	D1 I	Danko HR	Itaka × Glenlea	+	+	+	-
53.	D1 II	Danko HR	Itaka × Glenlea	+	+	+	-
54.	D1 III	Danko HR	Itaka × Glenlea	+	+	-	+
55.	D1 IV	Danko HR	Itaka × Glenlea	H	+	+	-
56.	D1 V	Danko HR	Itaka × Glenlea	-	+	+	+
57.	D2 I	Danko HR	Itaka × Glenlea	H	+	+	+
58.	D2 II	Danko HR	Itaka × Glenlea	+	+	-	-
59.	D2 III	Danko HR	Itaka × Glenlea	-	+	+	-
60.	D2 IV	Danko HR	Itaka × Glenlea	H	+	+	-
61.	D2 V	Danko HR	Itaka × Glenlea	+	+	+	-
62.	D3 I	Danko HR	Itaka × Glenlea	+	+	+	+
63.	D3 II	Danko HR	Itaka × Glenlea	-	+	+	-
64.	D3 III	Danko HR	Itaka × Glenlea	-	+	+	-
65.	D3 IV	Danko HR	Itaka × Glenlea	-	+	+	-
66.	D3 V	Danko HR	Itaka × Glenlea	H	+	+	+
67.	M × G I	Smolice HR	Merkawa × Glenlea	H	+	-	+
68.	M × G II	Smolice HR	Merkawa × Glenlea	H	+	+	+
69.	M × G III	Smolice HR	Merkawa × Glenlea	-	+	+	+
70.	M × G IV	Smolice HR	Merkawa × Glenlea	H	+	+	+
71.	M × G V	Smolice HR	Merkawa × Glenlea	H	+	+	+

+, presence; H, heterozygous.

**Table 2:** Evaluation of the level of infection development of tested hybrid forms of wheat.

Plant Name	Hybrid Form from BC_1_F_1_/F_2_ Generations	Level of Infection
H × G 1 I	(Harenda × Glenlea) × Harenda	8
H × G 2 III	(Harenda × Glenlea) × Harenda	6
H × G 3 IV	(Harenda × Glenlea) × Harenda	6
J × G 1 I	(Jutrzenka × Glenlea) × Jutrzenka	7
J × G 1 III	(Jutrzenka × Glenlea) × Jutrzenka	8
J × G 3 II	(Jutrzenka × Glenlea) × Jutrzenka	8
17 II	(Aura × Glenlea) × Aura	6
18 II	(Aura × Glenlea) × Aura	5
21 II	(Aura × Glenlea) × Aura	5
D1 III	Itaka × Glenlea	7
D3 I	Itaka × Glenlea	6
D2 V	Itaka × Glenlea	5
M × G II	Merkawa × Glenlea	6
M × G IV	Merkawa × Glenlea	5
M × G V	Merkawa × Glenlea	7

COBORU scale: 1—complete infection; 9—no infection.

**Table 3:** Average values of the obtained expression level (copies/μL) of the studied miRNAs, using the ddPCR method, for the studied genotypes at selected time points (hpi).

Hybrid Form	tae-miR9653b					tae-miR5384-3p				
	0 hpi	6 hpi	12 hpi	24 hpi	48 hpi	0 hpi	6 hpi	12 hpi	24 hpi	48 hpi
(Harenda × Glenlea) × Harenda	1149.51	1588.13	1340.06	1489.97	1726.36	89.77	84.14	146.39	167.93	154.10
(Jutrzenka × Glenlea) × Jutrzenka	425.41	434.56	475.90	836.50	362.28	129.60	62.57	120.24	147.38	130.92
(Aura × Glenlea) × Aura	741.59	1183.93	701.81	579.65	844.10	108.02	99.75	114.14	120.68	144.30
Itaka × Glenlea	1170.77	481.50	2169.09	1362.00	1332.23	102.19	86.76	103.48	131.42	160.96
Merkawa × Glenlea	774.80	738.21	2736.03	661.28	709.34	116.98	123.64	131.40	114.86	166.71

## Data Availability

The data presented in this study are available on request from the corresponding author.

## Supplementary Materials

The following supporting information can be downloaded at https://www.mdpi.com/article/10.3390/ijms26020665/s1.
