# Supplementary material for: Unraveling Effects of miRNAs Associated with APR Leaf Rust Resistance Genes in Hybrid Forms of Common Wheat (Triticum aestivum L.)
Source: Int J Mol Sci. 2025 Jan 14;26(2):665. doi: 10.3390/ijms26020665 (PMC11766205; doi:10.3390/ijms26020665)
Supplement: Supplementary file 1 [file ijms-26-00665-s001.zip › Supplementary Table S1.pdf]

Table S1. Statistical analysis of the *Lr34* gene for generations of BC_1F_1 and F_2 hybrid forms

Hybrid form of wheat	Time point	T/0h *Lr34*	Kolmogorov-Smirnov test (*Lr34*)	Levene's test (*Lr34*)	Student's t-test (*Lr34*)
**(Harenda × Glenlea) × Harenda**	00h		**0.55686**		
**(Harenda × Glenlea) × Harenda**	06h	1.48		0.3771	0.191489
**(Harenda × Glenlea) × Harenda**	12h	0.44		0.7633	0.015302
**(Harenda × Glenlea) × Harenda**	24h	1.17		0.2748	0.802742
**(Harenda × Glenlea) × Harenda**	48h	1.87		0.2334	0.214812
**(Jutrzenka × Glenlea) × Jutrzenka**	00h		**0.34987**		
**(Jutrzenka × Glenlea) × Jutrzenka**	06h	1.88		0.4265	0.611788
**(Jutrzenka × Glenlea) × Jutrzenka**	12h	1.00		0.4896	0.974766
**(Jutrzenka × Glenlea) × Jutrzenka**	24h	0.47		0.8512	0.184849
**(Jutrzenka × Glenlea) × Jutrzenka**	48h	1.55		0.2837	0.425004
**(Aura × Glenlea) × Aura**	00h		**0.21132**		
**(Aura × Glenlea) × Aura**	06h	0.50		0.8599	0.439384
**(Aura × Glenlea) × Aura**	12h	0.05		0.2739	0.101968
**(Aura × Glenlea) × Aura**	24h	0.48		0.5059	0.347336
**(Aura × Glenlea) × Aura**	48h	0.38		0.5083	0.268199
**Itaka × Glenlea**	00h		**0.55071**		
**Itaka × Glenlea**	06h	2.07		0.4979	0.3082
**Itaka × Glenlea**	12h	1.13		0.7392	0.875531
**Itaka × Glenlea**	24h	3.40		0.5382	0.173593
**Itaka × Glenlea**	48h	2.96		0.3546	0.189872
**Merkawa × Glenlea**	00h		**0.46751**		
**Merkawa × Glenlea**	06h	1.88		0.3998	0.416864
**Merkawa × Glenlea**	12h	1.28		0.7706	0.689807
**Merkawa × Glenlea**	24h	2.18		0.3758	0.458674
**Merkawa × Glenlea**	48h	2.39		0.601	0.289354
