# Supplementary material for: Unraveling Effects of miRNAs Associated with APR Leaf Rust Resistance Genes in Hybrid Forms of Common Wheat (Triticum aestivum L.)
Source: Int J Mol Sci. 2025 Jan 14;26(2):665. doi: 10.3390/ijms26020665 (PMC11766205; doi:10.3390/ijms26020665)
Supplement: Supplementary file 1 [file ijms-26-00665-s001.zip › Supplementary Table S2.pdf]

Table S2. Statistical analysis of the *Lr46-Glu2* gene for generations of BC_1F_1 and F_2 hybrid forms

**Hybrid form of wheat**	**Time point**	**T/0h *Lr46-Glu2***	**Kolmogorov-Smirnov test (*Lr46-Glu2*)**	**Levene's test (*Lr46-Glu2*)**	**Student's t-test (*Lr46-Glu2*)**
**(Harenda × Glenlea) × Harenda**	00h		**0.93508**		
**(Harenda × Glenlea) × Harenda**	06h	1.63		0.8474	0.297761
**(Harenda × Glenlea) × Harenda**	12h	1.36		0.9288	0.458419
**(Harenda × Glenlea) × Harenda**	24h	0.80		0.5644	0.61417
**(Harenda × Glenlea) × Harenda**	48h	0.64		0.803	0.432481
**(Jutrzenka × Glenlea) × Jutrzenka**	00h		**0.21316**		
**(Jutrzenka × Glenlea) × Jutrzenka**	06h	1.13		0.586	0.902172
**(Jutrzenka × Glenlea) × Jutrzenka**	12h	2.65		0.7879	0.320085
**(Jutrzenka × Glenlea) × Jutrzenka**	24h	0.30		0.4264	0.503999
**(Jutrzenka × Glenlea) × Jutrzenka**	48h	0.64		0.5387	0.732779
**(Aura × Glenlea) × Aura**	00h		**0.50442**		
**(Aura × Glenlea) × Aura**	06h	0.69		0.3215	0.715965
**(Aura × Glenlea) × Aura**	12h	0.20		0.3304	0.369011
**(Aura × Glenlea) × Aura**	24h	0.49		0.5321	0.578119
**(Aura × Glenlea) × Aura**	48h	0.52		0.4694	0.590078
**Itaka × Glenlea**	00h		**0.02397**		
**Itaka × Glenlea**	06h	6.60		0.1697	0.037973
**Itaka × Glenlea**	12h	1.25		0.9937	0.561017
**Itaka × Glenlea**	24h	1.28		0.8027	0.471528
**Itaka × Glenlea**	48h	4.31		0.4152	0.322773
**Merkawa × Glenlea**	00h		**0.13609**		
**Merkawa × Glenlea**	06h	2.80		0.9563	0.207233
**Merkawa × Glenlea**	12h	0.56		0.4129	0.630403
**Merkawa × Glenlea**	24h	0.41		0.5188	0.534022
**Merkawa × Glenlea**	48h	0.57		0.5434	0.65097
