# Supplementary material for: Unraveling Effects of miRNAs Associated with APR Leaf Rust Resistance Genes in Hybrid Forms of Common Wheat (Triticum aestivum L.)
Source: Int J Mol Sci. 2025 Jan 14;26(2):665. doi: 10.3390/ijms26020665 (PMC11766205; doi:10.3390/ijms26020665)
Supplement: Supplementary file 1 [file ijms-26-00665-s001.zip › Supplementary Table S3.pdf]

Table S3. Statistical analysis of the *Lr67* gene for generations of BC_1F_1 and F_2 hybrid forms

**Hybrid form of wheat**	**Time point**	**T/0h *Lr67***	**Kolmogorov-Smirnov test (*Lr67*)**	**Levene's test (*Lr67*)**	**Student's t-test (*Lr67*)**
**(Harenda × Glenlea) × Harenda**	00h		**0.81493**		
**(Harenda × Glenlea) × Harenda**	06h	1.50		0.3403	0.403446
**(Harenda × Glenlea) × Harenda**	12h	0.35		0.8376	0.010626
**(Harenda × Glenlea) × Harenda**	24h	1.99		0.3032	0.063052
**(Harenda × Glenlea) × Harenda**	48h	2.20		0.62	0.006616
**(Jutrzenka × Glenlea) × Jutrzenka**	00h		**0.64007**		
**(Jutrzenka × Glenlea) × Jutrzenka**	06h	1.24		0.7158	0.75961
**(Jutrzenka × Glenlea) × Jutrzenka**	12h	0.73		0.9928	0.690573
**(Jutrzenka × Glenlea) × Jutrzenka**	24h	0.60		0.5537	0.462844
**(Jutrzenka × Glenlea) × Jutrzenka**	48h	0.86		0.9565	0.832584
**(Aura × Glenlea) × Aura**	00h		**0.40764**		
**(Aura × Glenlea) × Aura**	06h	0.48		0.7212	0.32484
**(Aura × Glenlea) × Aura**	12h	0.05		0.185	0.063295
**(Aura × Glenlea) × Aura**	24h	0.90		0.5274	0.826662
**(Aura × Glenlea) × Aura**	48h	0.34		0.3603	0.173121
**Itaka × Glenlea**	00h		**0.52279**		
**Itaka × Glenlea**	06h	1.02		0.4934	0.968964
**Itaka × Glenlea**	12h	0.08		0.1742	0.140844
**Itaka × Glenlea**	24h	0.74		0.6585	0.686948
**Itaka × Glenlea**	48h	0.99		0.99	0.987796
**Merkawa × Glenlea**	00h		**0.2408**		
**Merkawa × Glenlea**	06h	1.21		0.9603	0.823572
**Merkawa × Glenlea**	12h	0.44		0.3915	0.442485
**Merkawa × Glenlea**	24h	0.35		0.5096	0.397643
**Merkawa × Glenlea**	48h	0.31		0.4033	0.357508
