# Supplementary material for: Unraveling Effects of miRNAs Associated with APR Leaf Rust Resistance Genes in Hybrid Forms of Common Wheat (Triticum aestivum L.)
Source: Int J Mol Sci. 2025 Jan 14;26(2):665. doi: 10.3390/ijms26020665 (PMC11766205; doi:10.3390/ijms26020665)
Supplement: Supplementary file 1 [file ijms-26-00665-s001.zip › Supplementary Table S4.pdf]

Table S4. Statistical analysis of reference genes (*ARF* and *TUBβ*) for BC1F1 and F2 generations of hybrid forms of wheat

Hybrid form of wheat	Time point	Cq_T-Cq-00h *ARF*	Cq_T/Cq_00h *ARF*	Kolmogorov-Smirnov test (*ARF*)	Levene's test (*ARF*)	Student's t-test (*ARF*)	Cq_T-Cq-00h *TUBβ*	Cq_T/Cq_00h *TUBβ*	Kolmogorov-Smirnov test (*TUBβ*)	Levene's test (*TUBβ*)	Student's t-test (*TUBβ*)	Student's t-test (*ARF* i *TUBβ*)
**(Harenda × Glenlea) × Harenda**	00h			**0.82206**					**0.92933**			
**(Harenda × Glenlea) × Harenda**	06h	2.437	1.10017816		0.2835	0.004629	0.043	1.00160059		0.4271	0.984892	0.245834
**(Harenda × Glenlea) × Harenda**	12h	1.397	1.05742086		0.9424	0.079174	-0.713	0.97365181		0.2794	0.15059	0.492119
**(Harenda × Glenlea) × Harenda**	24h	3.283	1.13498698		0.3808	0.036138	1.653	1.0610687		0.6103	0.111766	0.055094
**(Harenda × Glenlea) × Harenda**	48h	1.527	1.06276552		0.439	0.463383	-0.330	0.98781088		0.4582	0.801447	0.725251
**(Jutrzenka × Glenlea) × Jutrzenka**	00h			**0.88381**					**0.80447**			
**(Jutrzenka × Glenlea) × Jutrzenka**	06h	3.093	1.12977206		0.8944	0.004665	3.063	1.11453141		0.7962	0.033458	0.013233
**(Jutrzenka × Glenlea) × Jutrzenka**	12h	2.817	1.11816529		0.9386	0.004095	0.303	1.01134098		0.4006	0.734829	0.044479
**(Jutrzenka × Glenlea) × Jutrzenka**	24h	0.910	1.03817648		0.4934	0.301147	-0.263	0.99015454		0.4285	0.794314	0.621757
**(Jutrzenka × Glenlea) × Jutrzenka**	48h	3.987	1.16724934		0.7847	0.003213	2.633	1.09845464		0.5219	0.037289	0.0089
**(Aura × Glenlea) × Aura**	00h			**0.76827**					**0.86028**			
**(Aura × Glenlea) × Aura**	06h	1.903	1.07770822		0.6594	0.248269	0.483	1.01813634		0.3482	0.827636	0.524573
**(Aura × Glenlea) × Aura**	12h	2.017	1.08233533		0.4469	0.065814	0.590	1.02213884		0.9142	0.17851	0.086923
**(Aura × Glenlea) × Aura**	24h	3.437	1.14031029		0.4888	0.014027	1.873	1.07029393		0.5454	0.035244	0.017643
**(Aura × Glenlea) × Aura**	48h	3.847	1.15704954		0.4528	0.008856	1.703	1.06391495		0.7747	0.018233	0.010215
**Itaka × Glenlea**	00h			**0.38573**					**0.7523**			
**Itaka × Glenlea**	06h	1.673	1.06716618		0.504	0.191111	-0.220	0.99178594		0.7971	0.73636	0.44449
**Itaka × Glenlea**	12h	3.287	1.131924		0.464	0.003295	2.360	1.0881145		0.1649	0.003569	0.003155
**Itaka × Glenlea**	24h	4.650	1.18664704		0.5443	0.001005	3.213	1.11997511		0.6297	0.002147	0.001386
**Itaka × Glenlea**	48h	3.027	1.12148782		0.872	0.00975	-0.080	0.99701307		0.469	0.9485	0.132135
**Merkawa × Glenlea**	00h			**0.9393**					**0.60001**			
**Merkawa × Glenlea**	06h	1.517	1.06293223		0.2707	0.31308	0.167	1.00626174		0.8626	0.874271	0.512911
**Merkawa × Glenlea**	12h	4.077	1.16915629		0.8964	0.002154	1.847	1.06938009		0.9006	0.112308	0.016934
**Merkawa × Glenlea**	24h	2.883	1.11964039		0.569	0.025918	0.483	1.01815905		0.8898	0.59208	0.049843
**Merkawa × Glenlea**	48h	3.030	1.12572614		0.5345	0.015328	1.007	1.03782091		0.6166	0.269548	0.052514
